# In Vitro Study of the Role of Factor IX in Treatment of Breakthrough Bleeds in Patients with Hemophilia A and Inhibitors Receiving Emicizumab

**DOI:** 10.1055/a-2834-3178

**Published:** 2026-04-09

**Authors:** Maissaa Janbain, Laurie Josset, Alexandre Leuci, Karam Zakharia, Radia Ksayer, Christophe Nougier, Cindy Leissinger, Yesim Dargaud

**Affiliations:** 1 Section of Hematology and Medical Oncology, Deming Department of Medicine, Tulane University School of Medicine, New Orleans, Louisiana, United States; 2 Faculte de Medecine Lyon-Est, Universite Lyon I, UR4609, Hemostase and Thrombose, Lyon, France; 3 Unite d'Hemostase Clinique, Hospital Cardiologique Louis Pradel, Hospices Civils de Lyon, Lyon, France

**Keywords:** hemophilia A, inhibitors, emicizumab, factor IX, bypassing therapy

## Abstract

**Background:**

Hemophilia A (HA) is an X-linked disorder characterized by a deficiency of factor VIII (FVIII). Approximately 30% of patients with severe HA (baseline FVIII levels < 1 IU/dL) develop antibodies that neutralize the activity of FVIIIs. Emicizumab, a bispecific monoclonal antibody that has FVIII mimetic activity, is effective at preventing bleeding in patients with HA and has become a standard prophylactic therapy for hemophilia patients with inhibitors. However, breakthrough bleeding and perioperative management still require additional bypassing agents, which increases the risk of thrombosis in these patients.

**Methods:**

This in vitro study compares the hemostatic effect of adding factor IX (FIX) to the plasma of HA patients on emicizumab prophylaxis with the addition of bypassing agents. Blood from 24 HA patients on emicizumab was collected, and plasma samples were spiked with increasing concentrations of recombinant factor VIIa (rFVIIa), activated prothrombin complex concentrate (aPCC), recombinant FIX (rFIX), and plasma-derived FIX (pdFIX). Thrombin generation (TG) was assessed using calibrated automated thrombography. Plasma emicizumab and FIX activity were measured.

**Results:**

TG capacity improved significantly with increasing concentrations of rFVIIa ( *p* < 0.0001), aPCC, rFIX, and pdFIX ( *p* < 0.005). In contrast to high doses of aPCC and rFVIIa, TG parameters obtained with the addition of FIX did not cross the upper limit of normal physiologic ranges.

**Conclusion:**

Our data suggest a possible increased risk of thrombosis with higher concentrations of rFVIIa and aPCC, while adjunctive FIX therapy appears to be safe and effective in improving coagulation in HA patients with inhibitors on emicizumab prophylaxis. Future clinical trials are needed to confirm these results.

## Introduction

Hemophilia A (HA) is an X-linked disorder characterized by an inherited deficiency of factor VIII (FVIII). The clinical hallmark of this disorder is an increased bleeding tendency, which can be both spontaneous (in patients with severe deficiency) and related to trauma (in all patients), with hemarthrosis accounting for 90% of bleeds. Approximately 30% of patients with HA develop inhibitors to FVIII, making treatment with standard FVIII replacement therapy ineffective. 1 Until the approval of emicizumab, bypassing agents such as activated prothrombin complex concentrate (aPCC) and activated recombinant factor VII (rFVIIa) were the only approved therapies for the treatment and prevention of bleeding in HA patients with inhibitors. 2 The response to bypassing therapy is often unpredictable, variable, and difficult to monitor.

Emicizumab, while bridging activated factor IX (FIX) and factor X to activate FX, mimics the coagulant activity of FVIII, without being affected by the presence of FVIII inhibitors, thus offering an effective treatment for the prevention of bleeding in patients with (or without) FVIII inhibitors. 3 In the HAVEN 1 trial, emicizumab prophylaxis was associated with a significantly lower rate of bleeding events when compared with no prophylaxis among participants with HA with inhibitors (2.9 vs. 23.3 bleeding events per year, an 87% reduction in bleeding in patients on emicizumab [ *p* < 0.001]). 4 Early in the HAVEN one clinical trial, cases of thrombotic microangiopathy (TMA) and thrombotic events were reported in five patients who had bleeding events treated with large or repeated doses of aPCCs (an average cumulative amount of > 100 U/kg/24 hours for 24 hours while receiving emicizumab, leading to the restriction of the combination of emicizumab and aPCC if possible). 4 Currently proposed strategies for treating breakthrough bleeds or perioperative management in patients with FVIII inhibitors on emicizumab include rFVIIa and, when done under careful supervision, lower doses of aPCC. 5 In addition to the potential thrombotic risk of bypassing agents, they can lead to unpredictable responses, be difficult to monitor, and exhibit variable half-lives. Data from the U.S. Food and Drug Administration's Adverse Event Reporting System showed that thrombotic adverse events were three times more common with emicizumab than with FVIII products. Notably, 36.6% of these events were attributed to concomitant use of emicizumab with a bypassing agent. 6 Therefore, there is an increased need to develop an effective, relatively safe, and easily monitored adjunctive therapy for acute breakthrough bleeding and perioperative management of patients with HA and inhibitors while on emicizumab prophylaxis, particularly in elderly patients and those with thrombotic risk factors.

FVIII is the least abundant coagulation factor in plasma (approximately 1 nM). Given the higher levels of FIX (90 nM) and FX (135 nM), in physiological conditions, activated FVIII is therefore the limiting factor for activated factor X (FXa) formation. When used for routine prophylaxis, emicizumab concentration in plasma is around 0.37 µM. 7 As a result, the rate-limiting determinant for FXa generation is no longer FVIIIa mimetic, but the concentration of activated FIX (FIXa). One can therefore hypothesize that the addition of exogenous recombinant FIX concentrates containing a certain amount of activated FIX (rFIX) may improve FXa and thrombin generation (TG) in HA patients with emicizumab prophylaxis. 8 9

The objective of this proof-of-concept study is to evaluate the in vitro hemostatic effect of adding FIX to plasma samples from HA patients receiving emicizumab prophylaxis and compare it to the effect of traditional adjunctive therapies (rVIIa and aPCC) used to treat breakthrough bleeds in patients with HA and inhibitors.

## Methods

### Study Population

This study included 24 patients with severe HA, with or without FVIII inhibitors, receiving emicizumab prophylaxis. Eleven patients were recruited from the Tulane University comprehensive hemophilia care center in New Orleans (United States) and thirteen patients from the Lyon comprehensive hemophilia care center (France). All participants were recruited during a routine visit to their hemophilia center between November 2022 and June 2023 and provided informed consent. The study was approved by the local ethical committee of both Tulane University School of Medicine, LA (IRB 2022–200) and Hospices Civils de Lyons, France (CSE-HCL—IRB 00013204: 23_604). It was conducted in accordance with the Declaration of Helsinki.

All blood samples were taken at steady state; no patients were sampled during the loading phase of emicizumab prophylaxis. Twenty-two patients were on emicizumab prophylaxis at 1.5 mg/kg/week, and two were on 3 mg/kg every 2 weeks; none had received additional treatment. Spontaneous and traumatic bleedings after week 5 of emicizumab prophylaxis were recorded in the patients' clinical records. Twenty patients had no spontaneous bleeding during emicizumab prophylaxis, while four had spontaneous bleeding (one muscular, one soft tissue, one ankle joint, and one elbow joint). Patient characteristics are summarized in Table 1 .

**Table 1:** Demographic characteristics of our hemophilia A population treated with emicizumab

Demographic characteristics	Values
Age (y; mean ± SD)	34.2 ± 20.1 (range: 13–72)
Race	1 African American, 1 Hispanic, and 22 Caucasians
Regimen 1.5 mg/weekly, n (%)	22 (91.7)
Spontaneous breakthrough bleeds, n (%)	4 (17%)
Presence of inhibitor, n (%)	7 (29.2)
Baseline FIX levels	136.7 ± 59 IU/dL (range: 63.9–257.3)
Emicizumab drug levels	44.7 ± 16.1 µg/mL (range: 22.5–90.4)

TG, emicizumab quantification, and baseline FIX levels, using a one-stage test based on activated partial thromboplastin time (aPTT), were measured in all patients in the study.

### Blood Collection and Plasma Preparation

Blood samples were drawn into 2.7 mL tubes containing 0.106 M citrate for assay of FIX and emicizumab activity and into 3 mL tubes S-Monovette tubes (Sarstedt), containing 0.106 M trisodium citrate and 1.45 µM corn trypsin inhibitor for TG assay.

Platelet-poor plasma (PPP) and platelet-rich plasma (PRP) were prepared within 2 hours of blood collection. PPP and PRP were prepared according to the standardization recommendations published by the International Society on Thrombosis and Haemostasis. 10 Briefly, PPP was prepared by double centrifugation at 2,500 g for 15 minutes at 18°C. PRP was obtained by centrifugation at 150 g for 10 minutes at 18°C, and platelet count was adjusted to 150 × 10 ^9^ /L by dilution with the patient's own PPP.

### Thrombin Generation Assay

TG was measured in PPP and PRP samples using the calibrated automated thrombogram (CT, Stago, Asnière, France) and Thrombinoscope software (version 5.0.0.742), as previously described by Hemker et al. 11 PPP samples were triggered by using standardized reagent PPP-Low containing 1 pm TF and 4 µM phospholipids in final concentration (Stago) with 1.5 pm human activated factor XI (FXIa) in final concentration (Cryopep, Montpellier, France), and PRP samples by using standardized reagent PRP containing 1 pm TF (Stago). The synergic effect of emicizumab with BPA and rFIX or pdFIX was compared by spiking in vitro experiments. PPP samples were spiked with 0, 0.25, 0.5, 0.75, and 1.25 U/mL aPCC (FEIBA, Shire; equivalent to clinical doses of 0, 10, 20, 30, and 50 U/kg of aPCC) or 0, 25, 50, and 100 IU/dL FIX (rFIX, BeneFIX, Pfizer or pdFIX, AlphaNine, CSL Behring). PRP samples were spiked with 0, 1.125, 2.25, and 6.75 µg/mL of rFVIIa (NovoSeven, Novo Nordisk; equivalent to clinical doses of 0, 45, 90, and 270 µg/kg of rFVIIa). Briefly, for assay in the PPP well, 5 µL of PPP-reagent Low, 5 µL of a, and 10 µL of aPCC or rFIX or pdFIX were added to 80 µL of the PPP samples. And for TG in PRP, well, 10 µL of PRP reagent and 10 µL of rFVIIa were added to 80 µL of the PRP samples. All conditions were calibrated using a calibrator well containing 20 µL thrombin calibrator (Stago) added to 80 µL PPP or PRP and performed in duplicate. The addition of FXIa to PPP is based on previous studies that have shown a strong correlation between low TG values and spontaneous bleeds in patients on emicizumab. 12 13 All TG measurements were performed in duplicate.

Overall, the study comprised 22 independent experimental runs. Two patients presented on the same morning, and their samples were therefore analyzed within the same experimental session.

Only the endogenous thrombin potential (ETP), corresponding to the area under the thrombogram curve, was used to compare conditions, as it is considered a more comprehensive and sensitive marker of overall hemostatic capacity, accounting for both the amount and duration of TG, and this was correlated with clinical outcomes in patients with hemophilia and acquired hemophilia. 13 14

### Emicizumab Quantification and FIX Activity

Emicizumab quantification was performed by using a modified one-stage aPTT-based FVIII activity assay (mOSA) in the Lyon laboratory. The mOSA is calibrated with emicizumab-specific calibrators (R2 Diagnostics, South Bend, Indiana, United States) as previously described. 15 In Lyon, one stage-FVIII assay is performed using Synthasil reagent and HemosIL FVIII-deficient plasma (Werfen, Le Pré-Saint-Gervais, France).

Basal FIX levels were assessed in all patients at the Lyon laboratory using one-stage aPTT-based FIX activity. Briefly, diluted patient plasma was mixed 1:1 in HemosIL FIX-deficient plasma (Werfen, Le Pré-Saint-Gervais, France) and incubated for 3 minutes at 37°C with Synthasil reagent (Werfen, Le Pré-Saint-Gervais, France). The mixture is then recalcified, and the coagulation time is recorded. FIX activity levels were determined by comparing the clotting time measured to a standard curve. All analyses were performed on an ACL Top 750 analyzer using SynthasIL (Werfen, Le Pré-Saint-Gervais, France).

### Statistical Analysis

All data are represented as mean ± standard deviation (SD). ETP values were compared in each condition using a nonparametric one-way ANOVA. The relationship between ETP and FIX levels was assessed using Spearman's rank correlation test. Differences were considered to be significant when the *p* -value was < 0.05. Analysis was performed with GraphPad Prism (Version 4.3.0).

## Results

### Patient Characteristics

The mean plasma concentration of emicizumab was 44.7 ± 16.1 µg/mL (range: 2.2–90.4) and was within the recommended range. The mean baseline FIX level was 136.7 ± 59 IU/dL, ranging from 49 to 257 IU/dL, suggesting nondeficient FIX activity in our population ( Table 1 ).

### TG in Control Subjects and Emicizumab Patients

In our hands, the assay shows an intra-assay coefficient of variation of approximately 5% and a between-day coefficient of variation of approximately 10%. TG was assessed in a male control subject population ( *n* = 19) to obtain reference values for TG normalization in PPP and PRP samples. The basal hemostatic activity of patients treated with emicizumab was determined in PPP and PRP, based on the obtained ETP. In PPP samples, the mean ETP of severe HA patients receiving emicizumab was slightly lower than that of male control subjects (1,704 ± 273.8 nM minute vs. 2,334 ± 344 nM minute in control subjects), as was observed in PRP samples (1,169 ± 266.4 nM minute vs. 1,636 ± 262.8 nM minute in control subjects). Baseline TG was analyzed in relation to individual FIX levels. A significant positive correlation was observed between endogenous FIX concentration and TG capacity ( *r* = 0.562; *p* = 0.0065). These findings highlight that circulating FIX concentration, and consequently the amount of FIXa generated, is a key determinant of thrombin-generating potential in the presence of emicizumab.

### In Vitro Effect of BPA in the Presence of Emicizumab

**Fig. 1:**
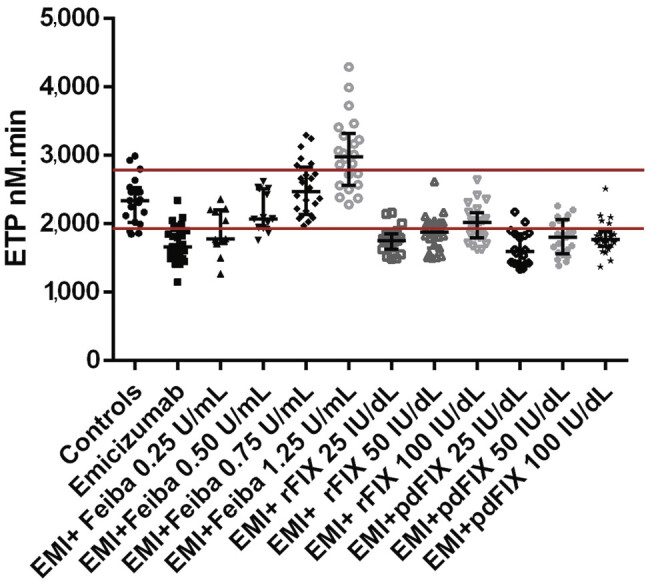
Endogenous thrombin potential (ETP) obtained with platelet-poor plasma (PPP) controls and PPP of patients on emicizumab at baseline and after spiking with different concentrations of aPCC (FEIBA), rFIX (Benefix), and plasma-derived FIX (Alfanine).

**Fig. 2:**
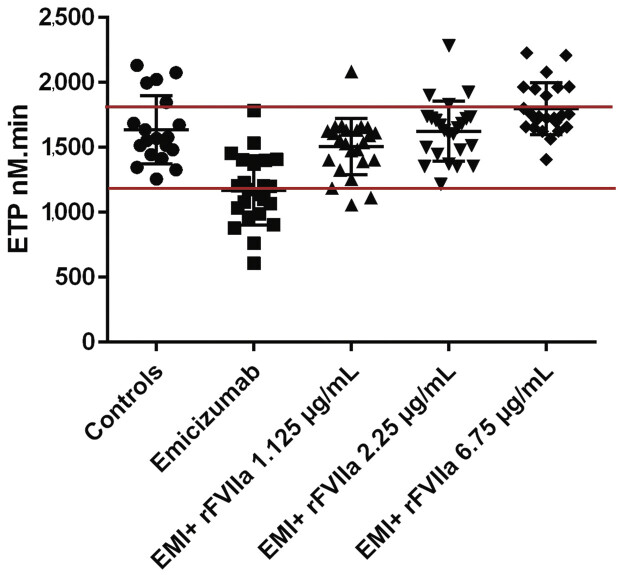
Endogenous thrombin potential (ETP) obtained with platelet-rich plasma (PRP) controls and PRP of patients on emicizumab at baseline and after spiking with different concentrations of rFVII.

Both aPCC ( Fig. 1 ) and rFVIIa ( Fig. 2 ) showed a concentration-dependent effect on TG in combination with emicizumab. The combination of aPCC at 0.5 U/mL with emicizumab resulted in normalization of TG (2,179 ± 294.4 nM minute; Fig. 1 ), as did the combination of rFVIIa at 1.125 µg/mL with emicizumab (1,506 ± 218.2 nM minute; Fig. 2 ). In contrast, the combinations of aPCC at 0.75 U/mL and 1.25 U/mL (2,516 ± 398.4 and 3,018 ± 526.3 nM minute, respectively; Fig. 1 ), and rFVIIa at 6.75 µg/mL (1,796 ± 199.7 nM minute; Fig. 2 ) with emicizumab resulted in an increase in TG above the upper limit of the normal range, suggesting a risk of hypercoagulability and thrombosis. Of note, the high dose of rVIIa 270 µg/kg (or equivalent in vitro spiking concentration of 6.75 µg/mL) was tested as it was historically used to control severe bleeding in patients with inhibitors, knowing that it is not recommended to use it in combination with emicizumab.

The comparison between the control group and aPCC at 1.25 U/mL using the Mann–Whitney test showed a significant difference ( *p* = 0.0018), indicating a clear shift toward hypercoagulability at this APCC dose. Likewise, rFVIIa at 6.75 µg/mL produced a highly significant increase ( *p* < 0.0001), further supporting its strong procoagulant effect under these assay conditions. The statistical significance was reached only for aPCC at 1.25 U/mL and for rFVIIa, but not for aPCC at 0.75 U/mL ( *p* = 0.32).

### In Vitro Effect of FIXa in the Presence of Emicizumab

Both rFIX (BeneFIX) and dpFIX (Alfanine) showed a concentration-dependent effect on TG in combination with emicizumab ( Fig. 2 ). ETP obtained after adding rFIX 100 IU/kg was significantly greater than that attained with pdFIX ( *p* = 0.027), which might be explained by the presence of the activated form of FIX in rFIX concentrates. Since FIX plays a role in the propagation phase of coagulation, contributing to amplification of TG, we examined the time-to-peak parameter, which better reflects propagation kinetics. At baseline, the mean time to peak was 6.0 ± 1.3 minutes. This parameter was shorter following rFIX supplementation (4.89 ± 1.3 minutes) compared with pdFIX (5.14 ± 1.4 minutes), supporting a faster thrombin burst with rFIX.

No significant difference was observed between the ETP of the different doses of dpFIX spiked into the emicizumab samples and the ETP of emicizumab alone. A significant difference for emicizumab + 100 IU/dL rFIX ( *p* = 0.02) was observed, but without normalizing TG.

**Fig. 3:**
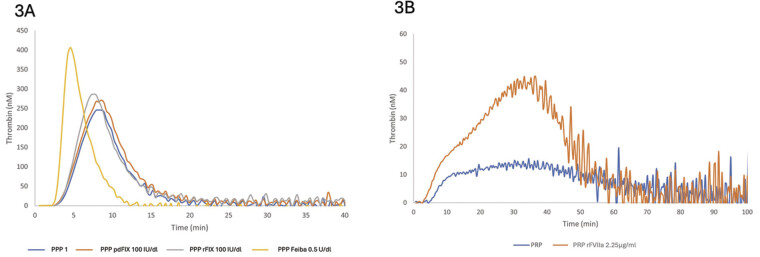
Thrombin generation profiles for a representative patient under various spiking conditions, with traces superimposed for comparison. ( **A** ) From left to right: emicizumab + pd FIX 100 IU/dL in green, emicizumab + rFIX 100 IU/dL in red, emicizumab + FEIBA 0.5 U/mL (20 IU/Kg) in yellow, and emicizumab alone in blue; all assessed in platelet-poor plasma (PPP). ( **B** ) From left to right: emicizumab with recombinant FVIIa (in vitro spiking dose rFVIIa 2.25 µg/mL or equivalent clinical dose of 90 µg/kg) in orange, then Emicizumab alone in blue, assessed in platelet-rich plasma (PRP).

TG profiles for a representative patient under various spiking conditions, with traces superimposed for comparison, are shown in Fig. 3 .

## Discussion

Emicizumab is a humanized bispecific monoclonal antibody that mimics FVIIIa, promoting activation of FX in the presence of activated FIX by bringing them in close proximity. Compared with FVIII, it provides more stable cofactor activity, has no on/off switch, and is immediately ready for action as soon as FIXa is available. 9 Its presence in molar excess shifts the rate-limiting control toward the amount of activated FIX. In addition to forming hemostatic ternary complexes with FIXa and FX, emicizumab also forms inefficient binary complexes with FIX, FIXa, FX, or FXa. Theoretically, the more ternary (FIXa-emicizumab-FX) complexes are available, the greater is the activation of FX and subsequently TG. The amount of the ternary complexes can be increased by infusion of exogenous FIX, preferably in its activated form, as commonly found in rFIX concentrates. This hypothesis can be effectively tested using the TG test, which is able to assess the overall coagulation capacity with the addition of a FIX concentrate or bypassing agents with emicizumab on board. 14 16 Our data confirmed the increase in TG capacity with the addition of increasing doses of FIX, notably with rFIX when compared with plasma-derived FIX that contains less activated FIX 17 ; acknowledging that in our study, we did not evaluate the specific contribution of aFIX in the concentrates, and more experiments will be needed to test this hypothesis. Indeed, our interpretation regarding the potential contribution of trace FIXa is based on published data 17 and should be considered a plausible mechanistic hypothesis rather than a directly demonstrated finding in our experimental material.

Moreover, the TG remained within physiologic normal ranges despite the addition of 100 IU/dL of FIX concentrate. This remains to be proven in prospective clinical trials where the safety and more durable efficacy of adding rFIX to emicizumab prophylaxis can be evaluated in patients with severe HA and inhibitors for treatment of breakthrough bleeds and perioperative management.

Our study shows increased TG, suggesting a potential risk of thrombosis, particularly at higher concentrations of rFVIIa and aPCC, which are commonly used in clinical management of patients. In a recent review by Koparkar et al at EAHAD 2023, 18 63 thromboembolic events were reported in patients receiving emicizumab and adjunctive factor therapy to increase TG. While the exact mechanism behind TMA remains elusive, a notable observation is that all cases of TMA have been documented in association with the use of aPCC at elevated concentrations. It is plausible to speculate that the co-administration of FXa and the two substrates (FIX and FX) of emicizumab present in aPCC may potentially play a role in precipitating these events. 19 More importantly, there were 57 thrombotic events noted, not related to the excessive use of aPCC. A deeper dive into this patient population, and after accounting for the use of devices or other cardiovascular risk factors, concerns were raised about the risk of using rFVIIa at higher doses. Indeed, a review of the EudraVigilance surveillance database revealed that one-third of the reported thrombotic adverse events occurred when rFVIIa concentrates were used concomitantly with emicizumab prophylaxis. 20 One hypothesis to explain the risk of thrombosis observed in patients treated with emicizumab is that, unlike activated FVIII, emicizumab is not inactivated by activated protein C (APC). This characteristic may contribute to a procoagulant shift and could partially explain the increased thrombotic risk observed when high doses of bypassing agents are administered. However, this mechanism cannot account for the markedly elevated TG induced by high concentrations of APCC and rFVIIa in our study. No APC was added to the assay, and in the absence of thrombomodulin, endogenous protein C cannot be activated. Therefore, APC-mediated effects cannot explain the pronounced increase in TG that we observe in our experiments.

These signals stress the importance of monitoring novel drugs in hemophilia, particularly when administered in association with bypassing agents. While rFVIIa remains the only recommended adjunctive therapy for treatment of breakthrough bleeds or perioperative management in patients with severe HA and inhibitors receiving emicizumab prophylaxis, one should be cautious with high doses. As shown in our study, thrombin potential surpasses the physiologic normal levels with greater doses of rFVIIa. A pattern that was not observed with the addition of FIX concentrates.

Early studies suggested that high plasma FIX levels may be a risk factor for venous thrombosis in the general population. 21 However, more recent studies have failed to demonstrate an association, despite being conducted in subjects with no underlying bleeding disorder such as FVIII deficiency. 22 Similar to our results, an in vitro TG study using a sequence-identical analog of emicizumab combined with a high concentration of purified human plasma-derived FIX 117 nM reported normalized coagulation capacity without a signal of hypercoagulability compared with normal controls (ETP = 1,196 ± 486 and 1,278.5 ± 329.7 nM minute, respectively). 19

In this small cohort of 23 patients, four experienced spontaneous breakthrough bleeding. Consistent with our previous findings, 12 their ETP was significantly lower compared with other emicizumab-treated patients who did not experience bleeding episodes. In addition, we had previously reported an association between normalization of TG results with bypassing therapy and the absence of surgery-related bleeding events. 14 Taken together, these TG findings, which are consistent with clinical outcomes of hemophilia patients, suggest that the enhanced TG observed in emicizumab-treated patients after in vitro addition of rFIX may represent an effective therapeutic strategy with reduced thrombotic risk. With the lack of better monitoring parameters, TG remains the best tool we can use to assess hemostasis from a global standpoint in patients with HA receiving emicizumab, especially when adjunctive therapy is used to augment hemostasis, taking into consideration the limitation of these tests, the variability of reagents used, and the potential for discordant results between in vitro and in vivo spiking experiments. 23 Indeed, TG normalization should not be interpreted as a therapeutic objective, and values approaching the normal range may warrant cautious interpretation depending on the trigger conditions used.

## Conclusion

In conclusion, our data suggest that FIX therapy may safely improve hemostasis in inhibitor patients receiving emicizumab prophylaxis. In addition to the advantage of monitoring levels with FIX therapy, the longer half-life of FIX compared with rFVIIa offers the potential advantage of controlling hemostasis with a single dose. Future prospective clinical trials are warranted to evaluate the efficacy and safety of this therapy in this patient population, with a particular focus on individuals over the age of 60 who are at higher risk for thrombosis.

## Notes

**What Is Known About This Topic?**

- In patients with inhibitors on emicizumab, breakthrough bleeds are treated with bypassing agents, with limited means to monitor and increased risk of thrombosis.
- In the presence of emicizumab, FIXa becomes the rate-limiting factor for thrombin generation, supplementation with recombinant FIX containing activated FIX may enhance hemostasis as a more predictable alternative to bypassing agents for managing breakthrough bleeding and perioperative care in hemophilia A with inhibitors.

**What Does This Paper Add?**

- In this in vitro study of plasma from hemophilia A patients on emicizumab, spiking with rFVIIa, aPCC, and FIX products showed dose-dependent increases in thrombin generation; however, FIX improved coagulation without exceeding physiologic ranges, unlike higher doses of rFVIIa and aPCC.
- These findings suggest that adjunctive FIX may provide a safer, effective, and more easily monitored option for hemostatic management in this setting, supporting the need for prospective clinical validation.
